# A Novel Protein Isoform of the Multicopy Human *NAIP* Gene Derives from Intragenic *Alu* SINE Promoters

**DOI:** 10.1371/journal.pone.0005761

**Published:** 2009-06-02

**Authors:** Mark T. Romanish, Hisae Nakamura, C. Benjamin Lai, Yuzhuo Wang, Dixie L. Mager

**Affiliations:** 1 Terry Fox Laboratory, BC Cancer Agency, Vancouver, British Columbia, Canada; 2 Department of Medical Genetics, University of British Columbia, Vancouver, British Columbia, Canada; 3 Cancer Endocrinology, BC Cancer Agency, Vancouver, British Columbia, Canada; 4 Department of Urologic Sciences, University of British Columbia, Vancouver, British Columbia, Canada; 5 The Prostate Centre, Vancouver General Hospital, Vancouver, British Columbia, Canada; Louisiana State University, United States of America

## Abstract

The human neuronal apoptosis inhibitory protein (*NAIP*) gene is no longer principally considered a member of the Inhibitor of Apoptosis Protein (IAP) family, as its domain structure and functions in innate immunity also warrant inclusion in the Nod-Like Receptor (NLR) superfamily. *NAIP* is located in a region of copy number variation, with one full length and four partly deleted copies in the reference human genome. We demonstrate that several of the *NAIP* paralogues are expressed, and that novel transcripts arise from both internal and upstream transcription start sites. Remarkably, two internal start sites initiate within *Alu* short interspersed element (SINE) retrotransposons, and a third novel transcription start site exists within the final intron of the *GUSBP1* gene, upstream of only two *NAIP* copies. One *Alu* functions alone as a promoter in transient assays, while the other likely combines with upstream L1 sequences to form a composite promoter. The novel transcripts encode shortened open reading frames and we show that corresponding proteins are translated in a number of cell lines and primary tissues, in some cases above the level of full length NAIP. Interestingly, some NAIP isoforms lack their caspase-sequestering motifs, suggesting that they have novel functions. Moreover, given that human and mouse *NAIP* have previously been shown to employ endogenous retroviral long terminal repeats as promoters, exaptation of *Alu* repeats as additional promoters provides a fascinating illustration of regulatory innovations adopted by a single gene.

## Introduction

Transposable elements (TEs) are ubiquitous components of most sequenced genomes, but their function, if any, is poorly understood. Comprising ∼50% of the human genome, the majority of TEs belong to the short interspersed element (SINE) (>10%), long interspersed element (LINE) (>20%), and endogenous retroviral/long terminal repeat (LTR) (∼10%) families [1]. The SINEs encode no open reading frame (ORF) and have utilized LINE-encoded proteins [2] to amplify to >10^6^ copies in the human and mouse genomes [1], [3]. On the other hand, only a limited number of LINEs and LTR elements are full-length; many of which are rendered non-functional due to point mutations and deletions [4]. Therefore, the majority of TEs no longer pose a significant burden as insertional mutagens, although many retain the regulatory signals necessary for transcription [5], [6].

The LTRs and LINEs naturally harbour RNA polymerase II (pol II) signals and numerous examples of promoter exaptation by host genes exist [5], [7], [8]. On the other hand, SINEs replicate via pol III [9], and thus are not expected to impose direct regulatory effects on protein-coding genes. Indeed, SINEs are over-represented within gene-rich regions, while the LTRs and LINEs are under-represented [6]. Recent scrutiny of the primate-specific *Alu* SINEs has provided various illuminating findings. They can be incorporated into mRNA as cassette exons [10], [11], and are often found in UTRs [8], [9], [12]. Furthermore, consensus binding motifs for many pol II transcription factors have recently been identified within *Alu*s [13], [14], but their role as promoters and enhancers has not been extensively researched.

We have previously shown that the neuronal apoptosis inhibitory protein (*NAIP*) orthologues in human (NM 022892.1) and mouse (NM 008670.2; NM 021545.1; NM 010870.2; NM 010872.2) provide a remarkable example of LTR promoter exaptation – unrelated LTRs were independently acquired as gene promoters [15]. *NAIP* is a member of the inhibitor of apoptosis protein (IAP) family, and was cloned as a candidate gene for the neurodegenerative disorder Spinal Muscular Atrophy (SMA) [16]. Consistent with its role as a modifier of SMA severity, *NAIP* has been shown to inhibit programmed cell death by binding activated caspases [17], [18], [19]. Moreover, the IAPs have emerged as therapeutic and diagnostic targets for various cancers [20], [21], [22]. Furthermore, the effect of NAIP expression in other neurodegenerative diseases, such as Alzheimer's disease, Down syndrome, multiple sclerosis, and Parkinson's disease, has also been investigated [23], [24]. Recently, a potential role in innate immunity surfaced through the discovery that polymorphism of a particular *Naip* copy in mouse strains determined permissiveness of *Legionella pneumophila* replication in host macrophages [25]. Paradoxically, *Naip*-mediated *L. pneumophila* restriction is caspase 1-dependent and signaling through this pathway results in the rapid death of infected cells [26], [27], [28]; a role consistent with its inclusion in the Nod-Like Receptor (NLR) superfamily of cytosolic pattern recognition sensors [29].

Here the flexibility associated with *NAIP* regulation in human is further demonstrated, by showing that 5′ truncated transcripts arise from two unique *Alu* SINEs. The resulting ORF is translated in a number of cell lines and primary tissues, and yields a protein possessing only the signature NLR domains. Since *Alu*s are over-represented in gene-rich regions and present transcription factor binding motifs, their role in establishing transcriptional networks is of great interest, as previously suggested [13], [30]. These findings indicate, for the first time, that *Alu* insertions can serve directly as gene promoters and derive novel transcripts and protein isoforms. The existence of NAIP protein isoforms, as described here, should therefore be considered in future experiments addressing its IAP and/or NLR functions.

## Results

### Human *NAIP* is a multicopy gene

**Figure 1:**
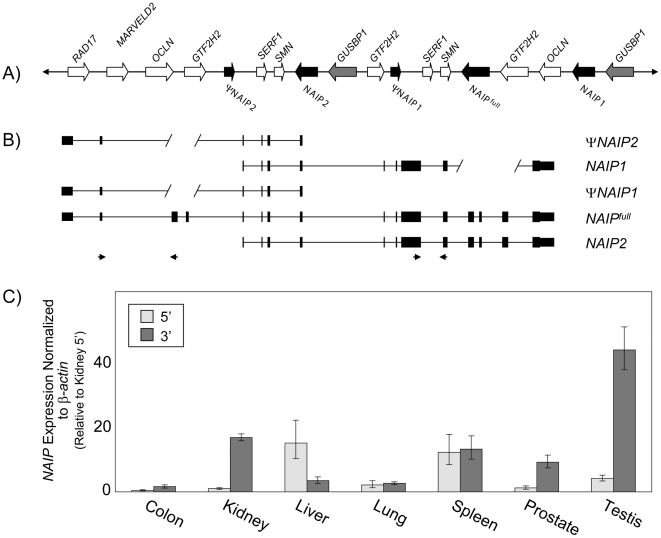
Expression of predicted *NAIP* copies in the sequenced human genome. A) General landscape of chromosome 5q13.2, including the *NAIP* (black arrows), *GUSBP1* (grey arrows), and surrounding genes (white arrows). B) Exon architecture of the annotated *NAIP* copies, verified by dot plots (Figure S1). Slanted lines delimit deletions relative to *NAIP^full^*. Diagrams are not drawn to scale. C) qRT-PCR with primers indicated by small arrowheads in panel B to determine the overall levels of *NAIP* 5′ (light bars) vs 3′ (dark bars) transcription. Values are normalized to β-actin levels in each tissue, and shown relative to kidney 5′. Each bar represents the mean of at least five independent experiments ± SD.

Copy number variation (CNV) exists in the region of human chromosome 5q13.2 encoding *NAIP* and other genes [31], [32], [33], as it does among inbred mouse strains [25]. In the reference human genome at least five copies are annotated [34] (Figure 1a), and while only one of these is full length, *NAIP^full^*, the others are assumed to be pseudogenes since two are 5′- and two are 3′-deleted, *NAIP1* & *2* and *ΨNAIP1* & *2*, respectively (Figure 1a, b). Exon content of the *NAIP* paralogues was verified using dot plots (Figure S1). While assessing their transcription using a variety of RT-PCR primers sets, we found that 3′ transcript levels of *NAIP* are greater than 5′ transcript levels in most tissues. In general, *NAIP* 5′ and 3′ transcripts showed the smallest differences in the macrophage-rich lung, spleen (Figure 1c), and blood (Figure S2). Expression of *NAIP* in these tissues most likely results from macrophage infiltration [35], the cell type mediating *NAIP-*dependent *L. pneumophila* immunity. The largest difference is observed in testis where 3′ levels are >40-fold above 5′ levels. Interestingly, in liver 5′ levels of *NAIP* are the highest (Figure 1c), potentially arising from transcription of 3′ deleted isoforms, premature poly-adenylation, or CNV-associated anomaly within the tissue sample screened. The abundance of 3′ transcripts raises the possibility that the 5′ deleted copies, *NAIP1* and *NAIP2*, are expressed (Figure 1c, Figure S2), or that internal promoters of *NAIP^full^* produce transcripts lacking the 5′ end, or both.

### Novel human *NAIP* transcription start sites

The observation that levels of 5′ vs. 3′ transcription are not uniform across various human tissues prompted an analysis to determine where *NAIP* transcription was initiating. Previously, we showed that an upstream ERV-P LTR is a promoter of *NAIP^full^* specifically in testis, but that ubiquitous expression derives from within an exon in the 5′ UTR [15]. Moreover, a previously published transcription start site [36], overlaps a MER21C LTR slightly upstream of the ERV-P, but could not be confirmed by 5′ RACE. However, an RT-PCR approach using tiled primers, similar to that of Xu et al. [36], indicated that an adjacent *Alu*Sx SINE was also included in these transcripts (Figure S3). We are unable to conclude whether this SINE is in fact a site of *NAIP* transcription or an internal exon of an undescribed 5′ UTR.

**Figure 2:**
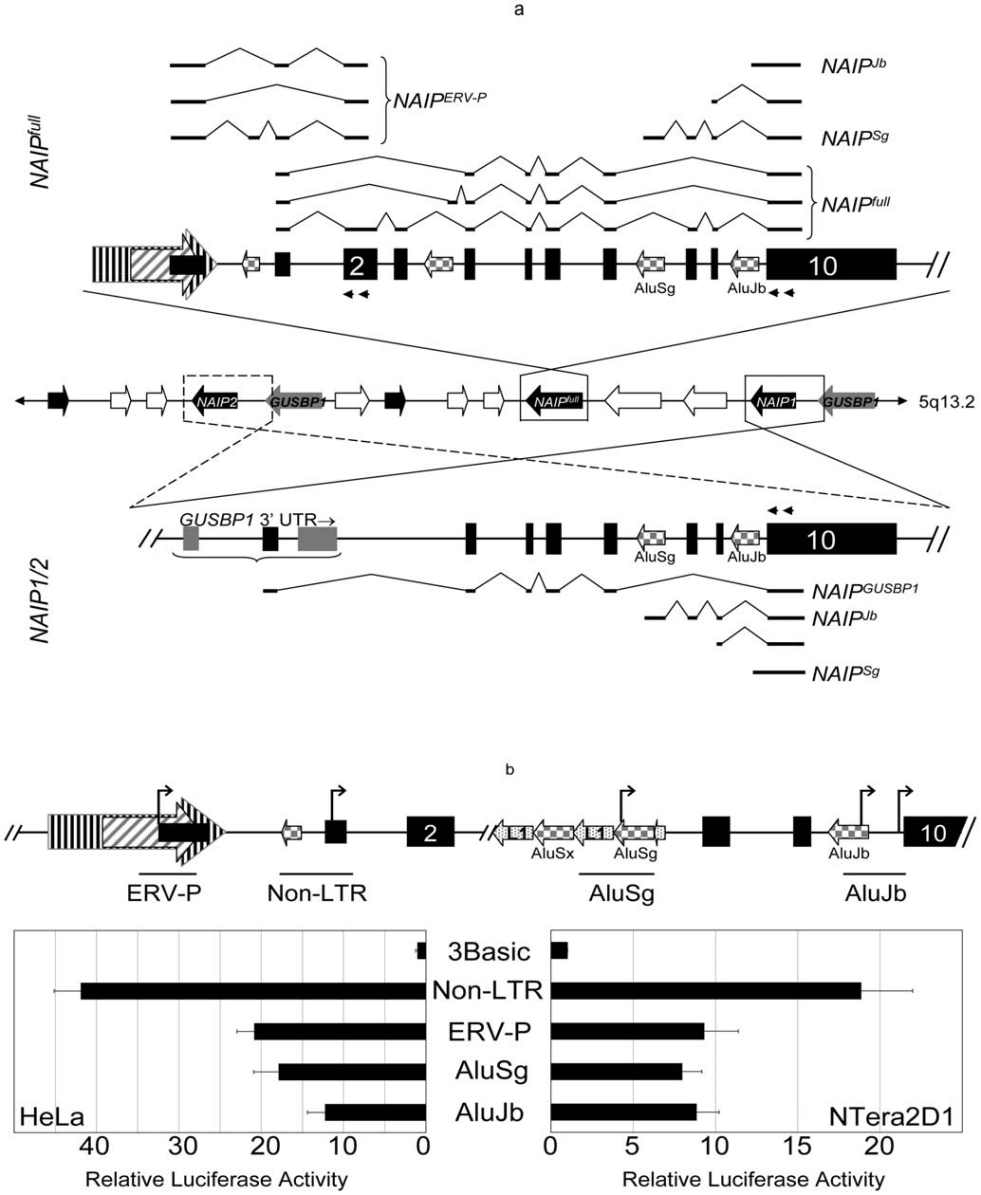
Identification of novel *NAIP* transcription start sites. A) Diagram of transcription start sites identified in the *NAIP^full^* (top) and *NAIP1/2* (bottom) copies by 5′ RACE. In the center, shaded block arrows indicate polarity of genes encoded on 5q13.2 (as in Figure 1a) and enlargements of *NAIP^full^* and *NAIP1/2* are shown above and beneath this representation. Their orientation is shown opposite to which they are encoded and black boxes represent exons. Checkered and striped block arrows indicate localization and orientation of *Alu*s and the previously identified *NAIP* LTR promoters [15], respectively. Not all repeat elements are shown. Black double arrowheads represent primers used in nested RT-PCR to uncover *NAIP* TSS in this and a previous analysis [15], represented by stick diagrams in top- and bottom-most images. All sequenced clones arising from *Alu*s, and neighboring TSS, map with perfect identity to *NAIP^full^*, *NAIP1*, and *NAIP2*. B) Novel regulatory regions associated with NAIP transcription. Luciferase assays were performed using reporter constructs centered on the previously identified ERV-P and *NAIP^full^*, and the *NAIP^Sg^* and *NAIP^Jb^* TSS identified here (indicated by bent arrows). The fragments tested are denoted by solid bars beneath the magnified *NAIP^full^* image (top), and are labeled accordingly. Exons, *Alu*s, and LTR elements are indicated as in Figure 2a; here, LINE fragments are indicated as speckled arrows. Values are normalized to an internal control (*Renilla* luciferase) and expressed relative to a promoter-less control vector (pGL3-Basic). Each bar represents the mean of at least four independent experiments ± SD. Gene diagrams are not drawn to scale.

Here we revised our previous 5′ RACE approach, which only assessed the transcription start sites (TSS) associated with expression of *NAIP^full^* [15], and numerous novel TSS were discovered (Figure 2). Unexpectedly, we observed that two *Alu* SINEs localized 5′ of exon 10, an *Alu*Sg and *Alu*Jb, are sites of *NAIP* transcriptional initiation, hereon referred to as *NAIP^Sg^* and *NAIP^Jb^* (Figure 2a). These *Alu*s are in the antisense orientation, full-length (∼300 bp) and present in *NAIP* orthologues of New and Old World primates (data not shown). Since sequence identity hinders their unambiguous mapping, *NAIP^Sg^* and *NAIP^Jb^* 5′ RACE clones could arise from three of the five copies (*NAIP^full^*, *NAIP1*, and *NAIP2*) in the reference human genome (Figure S4). Thus, either *NAIP1* and/or *NAIP2* are expressed from *Alu*s, or these *Alu*s may serve as promoters within *NAIP^full^*, or both.

A number of *NAIP^Sg^* clones were obtained that mapped to two distinct TSS localizing in the 3′ terminus of the *Alu* (Figure S4a). Interestingly, the *Alu*Sg A-rich tail is known to be hypermutable [37], [38], however, the corresponding region of this particular element is identical to its consensus sequence. The upstream ∼9 kb (relative to *NAIP^Sg^* polarity) is a patchwork of LINE fragments and *Alu*s, and likely contributes additional regulatory signals. All *NAIP^Sg^* clones splice into the adjacent exon 8 (Figure 2a, Figure S4a), utilizing a splice donor site frequently employed by exonized antisense *Alu*s [10], [11]. Several *NAIP^Jb^* clones were also obtained, these map to two particular regions localized near the *Alu*Jb 5′ terminus (Figure S4b). The regulatory signals comprising the *NAIP^Jb^* core promoter, therefore, are expected to lie within the body of this *Alu*. The *NAIP^Jb^* clones, however, do not splice into the downstream exon 10, rather transcription continues through the intervening ‘intron’. The validity of *NAIP^Jb^* transcripts is verified by +/− RT controls (Figure S5). Interestingly, the splice donor sequence utilized by *NAIP^Sg^* has undergone an AG→AT transversion mutation in *NAIP^Jb^* (Figure S4b); its capacity for splicing has not been studied here. Additional TSS downstream of *NAIP* ^Jb^, in the intervening sequence adjacent exon 10, are also observed (Figure S4b).

Another site of transcription initiation was identified within the final intron of the *GUSBP1* gene (Figure 2a). Although sequence identity hinders unambiguous mapping of this transcript, the novel first exon splices into exon 4 of the adjacent *NAIP1* and/or *NAIP2*. Consequently, expression of at least one other *NAIP* copy, in addition to *NAIP^full^*, is demonstrated since a TSS within the final intron of the *GUSBP1* gene is only adjacent to *NAIP1* and *NAIP2*.

### Promoter activity of proximal *NAIP^Sg^* and *NAIP^Jb^* sequences

Particularly intrigued by the *Alu* TSS, we tested the capacity of the underlying sequences as pol II promoters in reporter gene assays, relative to the 5′ promoters we previously identified [15]. Indeed, the ubiquitous *NAIP^full^* and LTR-derived, testis-specific *NAIP^ERV-P^* are capable promoters in the NTera2D1, HeLa (Figure 2b), and Jeg3 (data not shown) cell lines. A >500 bp DNA fragment underlying the *NAIP^Jb^* TSS, including the ∼200 bp of upstream *Alu* sequence and extending 5′ toward exon 10, exhibits strong promoter activity (Figure 2b). Similarly, a 600 bp fragment centered on the *NAIP^Sg^* TSS, containing the entire *Alu*Sg and the upstream 300 bp of internal L1 sequence, also exhibits considerable promoter activity relative to an empty vector control, in fact comparable to the LTR (Figure 2b). Due to location of the *Alu*Sg TSS, the upstream L1 fragment likely contributes promoter regulatory motifs, but its position relative to a full-length L1 does not correspond to the previously described antisense L1 promoter [7]. Analysis of the nucleotide sequences underlying the *NAIP^Sg^* and *NAIP^Jb^* TSS revealed the incidence of several putative pol II regulatory motifs, including: TATA-like boxes, initiator sequences, and downstream promoter elements (Figure S4) [39]. Accumulating evidence indicates that numerous pol II transcription factor binding sites lie within *Alu* elements [13], [14]. Indeed, both *NAIP*-associated *Alu*s possess potential AP-1 and retinoic acid- and estrogen response element binding motifs (Figure S4a,b), in agreement with published consensus sequences [13].

### Variable contribution of *Alu*-associated *NAIP* transcripts in different tissues

**Figure 3:**
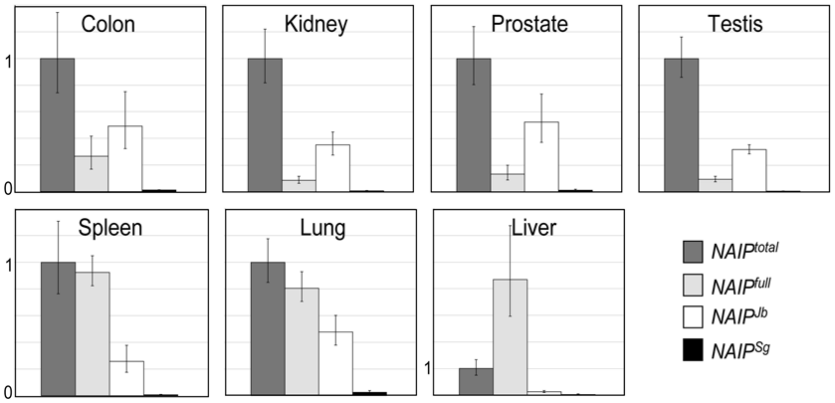
Contribution of *Alu*-initiated isoforms to total *NAIP* transcription. Expression levels of the targets: *NAIP^Total^* (3′), *NAIP^full^* (5′), *NAIP^Jb^*, and *NAIP^Sg^* were normalized to β-actin and are shown relative to 3′ levels of *NAIP* transcription in the indicated tissues. Each bar represents the mean of at least five independent experiments ± SD.

To address the contribution of *Alu*-derived *NAIP* transcripts to total *NAIP* expression, qRT-PCR was performed. Although their transcription is detected in most tissues screened by RT-PCR (Figure S5), this approach indicates *NAIP^Jb^* is expressed at levels similar to or higher compared to *NAIP^full^* in many of the tissues tested, and is therefore likely an important promoter (Figure 3). In contrast, *NAIP^Sg^* does not contribute significantly to total *NAIP* expression in any tissue tested (Figure 3). Interestingly, scrutiny of 5′ RACE sequences revealed that *NAIP^Sg^* undergoes RNA editing in its 5′ UTR (Figure S4a), a common observation among transcribed *Alu*s [40], [41]. Comparison of edited vs. un-edited *NAIP^Sg^* transcript levels indicated the former is >10-fold more abundant than the latter (data not shown).

Most *NAIP* transcription in colon, spleen, lung, and prostate could be accounted for by the combined activity of all queried promoters, but the contribution of individual paralogues could not be assessed due to their high sequence identity. However, in kidney and testis all isoforms are not detected and it is likely that unaccounted 3′ transcription either initiates downstream of *Alu*Jb, as indicated above (Figure S4b), or from the *NAIP^GUSBP1^* TSS. Contribution of *NAIP^GUSBP1^*-derived transcripts could not be assessed due to the complexity of alternative splicing in this 5′ UTR (Figure S5). As discussed previously, the 5′ levels of *NAIP* in liver are expressed 4-fold over 3′ levels, suggesting that all transcription in this tissue derives from *NAIP^full^*. Since two independent liver RNA samples were screened, this rules out the possibility of patient-specific CNV, unless both samples derive from the same patient. Perhaps transcription in liver produces isoforms that constitutively omit one or both exons to which our 3′ qRT-PCR primer sets are designed. Alternatively, *NAIP^full^* transcripts in this tissue could be aberrantly poly-adenylated. Regardless, neither *NAIP^Sg^* nor *NAIP^Jb^* are highly expressed in liver.

### Full-length *Alu*-derived transcripts are broadly expressed

**Figure 4:**
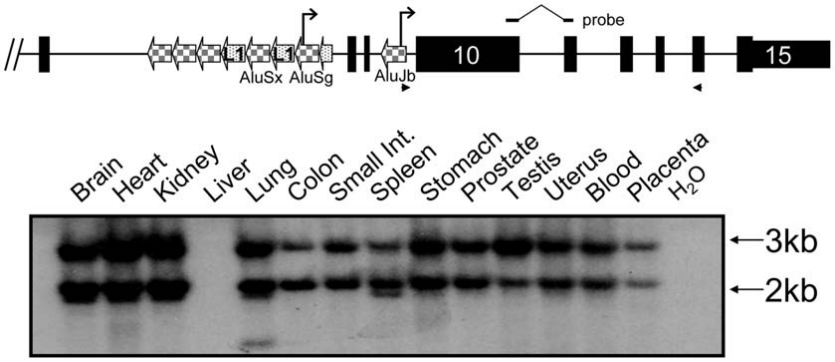
Expression of full-length *NAIP^Jb^* transcripts across many tissues. At top, a schematic diagram of the 3′ terminus of *NAIP* is shown, not to scale. Exons are indicated by black boxes, checkered and spotted arrows indicate the polarity of SINEs and LINEs, respectively. Not all repeat elements are shown. The arrowheads represent primers used to assess full-length *NAIP* transcription. Due to the high copy number of *Alu*s in the human genome, the resultant RT-PCR gels were resolved by Southern blotting, with the unique probe shown, across the indicated tissues to reveal true *Alu*Jb-derived *NAIP* transcripts.

The fact that the *Alu*Jb functions as a pol II promoter is an intriguing finding, with genome-wide ramifications in establishment of transcriptional networks, as previously suggested [13], [30]. We next examined the potential for transcription of a novel *NAIP* ORF as a result of *Alu* promoter activity. Indeed, if all downstream exons are included in at least some *Alu*-derived *NAIP* transcripts, a 2,643 nucleotide ORF is preserved (Figure S6). Therefore, we sought to determine whether *Alu*-initiated transcripts continue to the 3′ terminus, by RT-PCR. Southern blotting was required since, by necessity, primers hybridized to *Alu*s – the most plentiful elements in primate genomes [1]. Across all tissues screened, except liver, products corresponding to the expected size (∼3 kb) were resolved for *NAIP^Jb^* (Figure 4). Among various minor forms, one notable variant of ∼2 kb is expressed at the same frequency as full-length *NAIP^Jb^*. This ∼2 kb variant, among numerous others including full-length, is also observed for *NAIP^Sg^* transcripts in several tissues (data not shown). Potentially the smaller isoform could result from alternative splicing common to both *NAIP^Jb^* and *NAIP^Sg^* transcripts, between the site of reverse primer binding and probe hybridization. Alternatively, a single *NAIP* transcript possessing a second exonized *Alu* downstream of some or all of the probe-binding region could also explain this observation. The prominent ∼3 and ∼2 kb bands do not result from the simultaneous amplification of NAIP^Jb^ and NAIP^Sg^ due to primer cross-reactivity, since the respective transcripts and their unique 5′ UTRs are roughly equal in size. Nonetheless, existence of full-length *Alu*-derived transcripts, a potential 2,643 nucleotide ORF, and numerous in-frame ATGs in accordance with derived consensus sequences [42], [43] (Figure S6) suggest a potential for the synthesis of NAIP protein isoforms.

### Novel human NAIP protein isoforms

**Figure 5:**
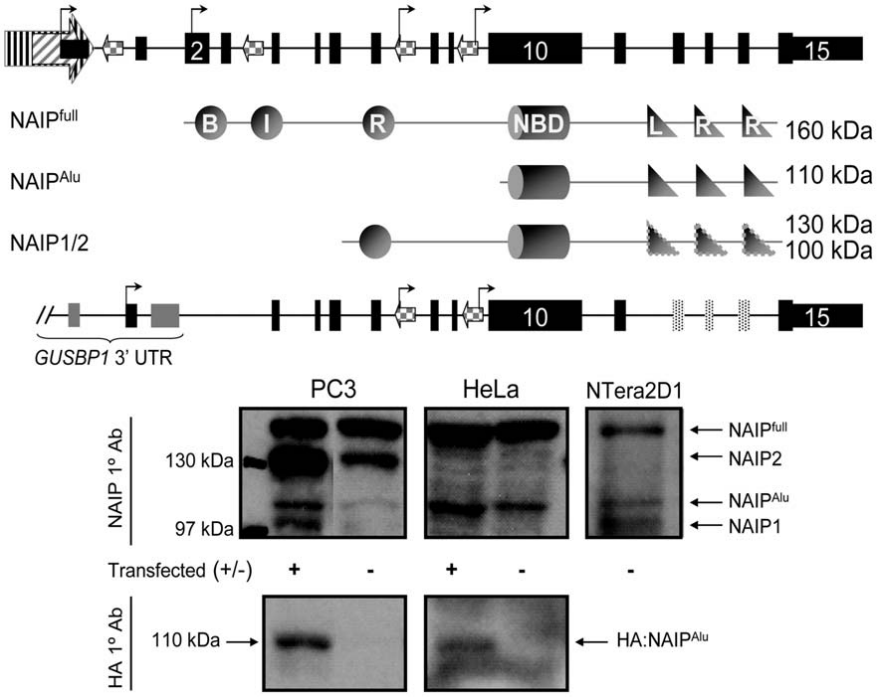
Detection of novel NAIP protein isoforms. A) Diagrams of *NAIP^full^* (top) and *NAIP1/2* (bottom) are shown; speckled exons 12–14 are only encoded by *NAIP2* in the reference human genome. The known *NAIP* TSS are indicated by bent arrows, and computational translation predicts the domain composition and mass of the resulting ORFs: NAIP^full^, NAIP*^Alu^*, NAIP^1/2^. NAIP^1^ is predicted to encode a ∼100 kDa protein, and NAIP^2^ is ∼130 kDa. The BIRs (Baculoviral IAP Repeat); NBD (Nucleotide binding domain) and LRR (Leucine-rich repeat) domains are indicated by circles, cylinders, and triangles respectively. B) Western blot of NAIP in PC3, HeLa, and NTera2D1. Endogenous expression of NAIP^full^, NAIP^2^, NAIP*^Alu^*, and NAIP^1^ (top) and HA-tagged NAIP*^Alu^* (bottom) is shown in transfected and untransfected cells.

Using the annotated copies of *NAIP* in the sequenced human genome as a reference [34], we scanned all possible full-length transcripts that could arise from the novel TSS reported above for ORFs and domain composition. Many potential ORFs were identified for each queried transcript, but only the longest examples were considered. Interestingly, all accepted examples represented N-terminal truncations of NAIP^full^, indicating the existence of numerous potentially functional in-frame translation initiation codons (Figure 5a, Figure S6). NAIP^full^ was previously shown to comprise 1403 amino acids and yield a ∼160 kDa protein encoding three N-terminal anti-apoptotic Baculoviral IAP Repeat (BIR) domains, followed by a central nucleotide binding domain (NBD) and C-terminal leucine-rich repeats (LRR) [16]. *NAIP^Sg^*- and *NAIP^Jb^*-mediated transcription of *NAIP2* is predicted to generate an ORF 881 amino acid long, and corresponds to a 110 kDa protein that excludes the BIRs (NAIP*^Alu^*). Due to the deletion of exons 12-14 in *NAIP1* a C-terminal truncation of the LRRs is also predicted, in addition to a truncation of its N terminus (Figure 1b), and could produce a ∼85 kDa NAIP protein isoform, but was not detected. Finally, transcription from the promoter within the final *GUSBP1* intron can drive expression of both *NAIP1* and *NAIP2*, and potentially gives rise to 100 kDa (NAIP1) and 130 kDa (NAIP2) proteins, respectively. Both putative protein isoforms, NAIP1 and NAIP2, possess one N-terminal BIR domain, followed by the central NBD, but only NAIP2 harbours C-terminal LRRs. Indeed, western blots on human PC3, HeLa, and NTera2D1 cell lysates indicate the presence of multiple bands corresponding to the above computer predictions (Figure 5b). To more accurately assess the potential for translation of the *Alu*-derived *NAIP2* ORF we generated a NAIP:hemagglutinin fusion protein (HA∶NAIP*^Alu^*) and over-expressed it in the cell lines indicated above. The recombinant protein HA∶NAIP*^Alu^* is translated and migrates at 110 kDa with the putative endogenous isoform (NAIP*^Alu^*) in untransfected PC3 and HeLa cells (Figure 5b). It is clear the NAIP protein isoforms are differentially expressed in the queried cell lines, but all three cell lines endogenously produce the ∼160 kDa NAIP^full^ and ∼110 kDa NAIP*^Alu^* proteins, albeit to a different degree. In the PC3 and HeLa cell lines, where HA∶NAIP*^Alu^* was overexpressed, an increase in band intensity is seen compared to NAIP*^Alu^* in untransfected cells. Overall, expression of the putative NAIP*^Alu^* protein is low relative to NAIP^full^ in all cell lines, however, the difference is not as exaggerated in NTera2D1 cells compared to PC3 or HeLa. Lastly, it appears that neither NTera2D1 nor HeLa cells express the putative ∼130 kDa NAIP2 protein isoform.

### NAIP protein isoforms are broadly expressed in human tissues

**Figure 6:**
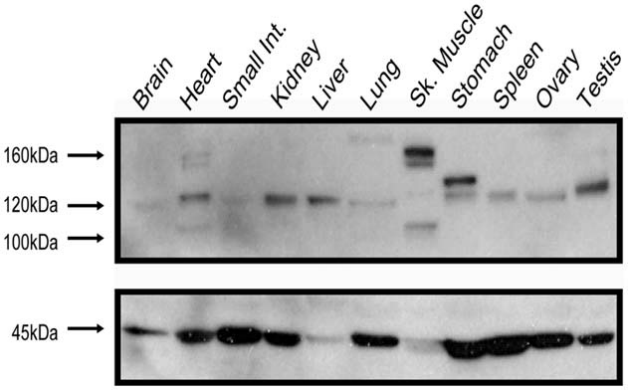
Expression of NAIP protein isoforms in primary human tissues. Western blot analysis of a commercial, pre-transferred membrane with human proteins deriving from the tissues of one adult female, with the exception of testis. NAIP expression is shown at top, and actin levels at bottom. Mass of bands is indicated at left.

The observation that NAIP proteins equivalent in size to all of the computer-predicted isoforms are expressed in the cell lines screened, prompted a similar investigation of primary human tissues (Figure 6). A variety of NAIP proteins were detected in most of the tissues examined, although NAIP^full^ is not broadly expressed. In fact, NAIP^full^ was only detected in heart, skeletal muscle, and at very low levels in testis. Similarly, the ∼110 kDa protein, which is expected to represent the *Alu*-derived *NAIP* ORF, is also only detected in heart and skeletal muscle. Potential NAIP2 proteins at ∼130 kDa are observed almost uniformly across the tissues tested, and could correspond to *NAIP^GUSBP1^*-initiated transcripts. The subtle variation of the putative NAIP2 proteins, such as in spleen and heart, could result either from alternative start codon selection (Figure S6) or alternative splicing of *NAIP2* terminal exons. Importantly, all of the tissues screened here, other than testis, derive from one individual with unknown *NAIP* copy number and mRNA expression levels. Nonetheless, we demonstrate the expression of various human NAIP protein isoforms that correspond with calculated molecular weights of the ORFs generated by alternative promoter usage.

## Discussion

Transposable elements were initially discovered as important factors in the regulation of gene expression in maize, and termed controlling units [44]. This view of TE usefulness was contrasted by the ‘junk DNA’ hypothesis [45]. In recent times their practicality has garnered increased attention, particularly as mobile regulatory modules [5], [9], [13], [30]. Strikingly, TEs are associated with many evolutionarily constrained regions in mammalian genomes [46], and many conserved non-coding elements are reported to function as transcriptional enhancers [47]. In general, it is difficult to ascertain the extent to which TEs donate their embedded regulatory signals to cellular genes, particularly because they can impose their effects over great distances. However, bioinformatics analyses of human and mouse genomes indicate a substantial impact of TEs on cellular gene regulation; as many as 25% of genes possess TEs in their UTRs [8], [48]. Therefore, their influence on increasing the diversity of mammalian transcriptomes is likely underappreciated.

The LTRs and LINEs, due to the natural presence of RNA pol II signals, are likely candidates to fulfill a regulatory role for cellular genes; dozens of known cases confirm their utility as regulatory modules [5], [7], [8]. In contrast, the pol III-dependent SINEs are concentrated in gene dense regions [1], [6], but have largely been neglected as modulators of cellular gene expression. Recent bioinformatics analyses, however, have revealed the presence of numerous RNA pol II transcription factor binding sites and hormone response elements within SINEs [13], [14], substantiating an earlier report [49]. Notably, the primate-specific *Alu*s – divided into the old *Alu*J, intermediate *Alu*S, and young *Alu*Y subfamilies – present consensus transcription factor binding sites distributed in an age-dependent manner [13]. Interestingly, among all gene-associated *Alu*s on chromosome 21 and 22, older elements tend to harbour estrogen response elements and AP-1 docking sites, while younger and/or polymorphic *Alu*s are enriched for other features, including retinoic acid response elements. In addition, important roles in mRNA poly-adenylation have also been revealed for *Alus* and other TEs in a variety of organisms [50], [51]. Since *Alu*s number >10^6^ copies in the human genome, are enriched in gene-dense regions, and contain potential pol II transcriptional regulatory motifs, they could be considered the most important transcriptional regulators.

For the first time it is shown here that an *Alu* can function as a direct promoter for a human gene. More commonly, they and other SINEs are incorporated into mRNA UTRs and coding regions as cassette exons [5], [8], [9], [52], facilitated by the presence of numerous splice donor and acceptor sites in the sense and antisense orientations [10]. Examples of SINE exaptation as promoters, however, are limited and represented by a sense B1 [53] and an antisense B2 [54] element in mouse. In human, an isoform of the *p75TNFR* gene initiates transcription from an antisense MIR SINE, with the adjacent *Alu*Jo providing an alternative translation start site [55]. Furthermore, a bioinformatics analysis reports the existence of several unvalidated antisense *Alu*-associated TSS [8]. Here, broad transcription of *NAIP* isoforms from exapted antisense *Alu*Jb and *Alu*Sg elements is demonstrated in a number of tissues, but it is unknown whether these sequences would also be functional in the sense orientation. The Sg and Jb exaptations associated with *NAIP* transcription belong to older families that exhibit 10% and 15% divergence from their consensus sequences, respectively. Remarkably, *NAIP^Jb^*-associated transcripts are more highly expressed than full-length isoforms in many tissues, but *NAIP^Sg^* levels are at the limit of detection. We further demonstrate that the *Alu*-initiated *NAIP* transcripts extend to the 3′ terminus, and that the associated ORF, harbouring only NBD and LRRs, is translated in a variety of cell lines and primary human tissues. Our findings also suggest that the other predicted novel NAIP proteins are expressed, in addition to the BIR-less isoform directly assessed here. It is notable that the tissue blot we screened derives from one adult individual, with the exception of testis, indicated by the manufacturer as an accidental fatality. An earlier analysis of pooled primary human tissue samples using a different antibody, also revealed similar NAIP protein isoforms that were speculated to arise by alternative splicing [35]. Nonetheless, the data presented here substantiate transcriptome analyses that reveal alternative promoter usage as an important source of alternative mRNAs and proteins [56], [57].

The *NAIP* gene first rose to prominence when it was cloned as a putative disease allele for the neurodegenerative disorder, Spinal Muscular Atrophy (SMA) [16], but is now understood to influence SMA severity, which is induced by the adjacent SMN gene [58]. Its identification did seed discovery of the Inhibitor of Apoptosis Protein (IAP) family in animals [19]. The IAPs sequester activated caspases, the agents of cell death, via their signature N-terminal BIR domains [20]. Interest in *NAIP* was renewed through the discovery that polymorphism of the murine *Naip5* (*Birc1e*) copy solely determines permissiveness of *Legionella pneumophila* replication in host macrophages [25]. Human *Legionella* infections result in Legionnaire's disease, a severe type of pneumonia [59]. It was recently shown that human NAIP also blocks *L. pneumophila* replication in cell lines and primary cells, suggesting a common function [60]. NAIP-dependent sensing of cytosolic microbial patterns is LRR-dependent, and is currently known to respond to *Legionella* and *Salmonella typhimurium* flagellin [26]. These and other findings point to an important role in the innate immune response, and justify the inclusion of NAIP in the NLR superfamily [29]. Invariably, the NLRs possess a central NBD and C-terminal LRRs; collectively they survey the cytosol for pathogen associated molecular patterns and elicit the appropriate response [61].

While the potential functions of the novel NAIP protein isoforms are unknown, there are several possibilities. Firstly, NAIP proteins are known to homo-oligomerize via their NBD [17], therefore, expression of BIR-truncated isoforms and their subsequent interaction with NAIP^full^, could be a mechanism whereby its anti-apoptotic properties are effectively dispersed among a greater number of cytosolic molecules. Alternatively, these could be dominant negatives and serve to regulate the amount of anti-apoptotic NAIP molecules active in a given cell. Finally, expression of NAIP protein isoforms could represent a new example of innovation within the innate immune system, whereby hetero-oligomerization of NLRs creates diversity among these cytosolic sensors, analogous to the Natural Killer inhibitory cell receptor repertoire [62]. Indeed, NBD-mediated heterotypic interactions of some NLRs, including NAIP, have been demonstrated [63]. Moreover, Naip was also shown to co-precipitate with its closest homologue, ICE protease activating factor (Ipaf) [27]. Together these proteins activate Interleukin converting enzyme (ICE or caspase 1), and initiate caspase 1-dependent cell death in response to cytosolic flagellin [26], [27], [28]. Although caspase 1 is required to cleave the inflammatory cytokines proIL-1β and proIL-18 into their active forms, their involvement in this process remains unresolved. Interestingly, and perhaps not coincidentally, the cellular processes affected by IL 1β – proliferation, differentiation, and apoptosis – are the same as those influenced by AP-1 transcriptional regulation [64].

Genes involved in immunity tend to permit regulatory variation [8], as do multicopy genes [52]. While it is known that alternative 5′/3′ ends create genetic variation that leads to proteome evolution [56], [57], [65], the effect of *Alu* elements is under appreciated. Here we show that transcription from *Alu*s generates a novel *NAIP* ORF that is subsequently translated, clearly indicating the effect they have on not only gene regulation, and perhaps establishment of transcriptional networks [13], [30], but also proteome evolution.

## Methods

### Ethics Statement

The blood sample was obtained with written informed consent according to a protocol approved by the University of British Columbia Research Ethics Board.

### RNA and Reverse Transcription

With the exception of blood, all human RNA was purchased from Clontech (Mountain View); each sample consists of pooled material from multiple individuals. Blood was obtained from a healthy human adult with informed consent and the sample subsequently underwent erythrocyte reduction. RNA from remaining peripheral blood leukocytes (PBLs) was isolated using the QIAmp RNA Blood Mini Kit (Qiagen). Where necessary, RNA was isolated from candidate cell lines using TRIzol (Invitrogen) according to the manufacturer's recommendations. Prior to reverse transcription, RNA was quantified using a Qubit fluorometer (Invitrogen). All cDNA synthesis was prepared by random hexamer-primed Superscript III Reverse Transcriptase (Invitrogen), as directed by the manufacturer.

### RT-PCR

All RT-PCR, except as indicated below for amplification of the *NAIP* ORF and generation of the expression vector, was performed with Platinum Taq DNA Polymerase (Invitrogen) and the relevant primers are listed in Table S1, all used at 10 µM. Optimal primer annealing temperatures were deduced using the temperature gradient function of an iCycler (Bio-Rad) over 35 cycles. Subsequent experiments were carried out at the optimal T_m_ for each primer set in a GeneAmp PCR System 9600 (Applied Biosystems). Discrimination of 5′ vs 3′ *NAIP* transcript levels was carried out at 30 cycles. The full-length *NAIP* ORF deriving from the *Alu* SINEs was obtained by amplification with Phusion High Fidelity DNA Polymerase (Finnzymes). As expected, primers within *Alu* SINEs yielded a multitude of products and were subsequently resolved by Southern blotting. Probe was generated with radiolabeled dCTP^32^ using the random primer labeling kit (Invitrogen) as directed. Pre-hybridization, hybridization, and washes of Zeta-probe GT membranes (BioRad) were performed using ExpressHyb (Clontech) according to manufacturer's specifications. Exposure of BioMax Film (Kodak) for one hour or less was sufficient to adequately differentiate true bands from background.

### 5′ Rapid Amplification of cDNA Ends

Using the First-choice RLM RACE Kit (Ambion) the 5′ termini of human *NAIP* were deduced as before [15]. We revised our initial approach [15] by designing gene-specific reverse primers to a downstream exon, common to all predicted *NAIP* copies (primers listed in Table S1); previously primers could only surmise expression of *NAIP^full^*. Subtle variations in RT-PCR product size was observed across a range of T_m_s (55°–60°) – since the full complement of *NAIP* start sites was being queried – therefore, all unique bands were purified using the QIAquick Gel Extraction Kit (Qiagen) and cloned into the pGEM-T vector (Promega) prior to sequencing (McGill University and Génome Québec Innovation Centre). Importantly, consistent amplification patterns were observed within a given T_m_. We similarly tested mouse kidney RNA; although we identified novel intraexonic start sites for *mNaip2*, qRT-PCR only showed a slight increase (1.2∶1) of 3′ over 5′ ends (data not shown).

### Quantitative RT-PCR

The cDNA used for quantitative RT-PCR with Power SYBR Green PCR Master Mix (Applied Biosystems) in the ABI 7500 Real Time PCR System (Applied Biosystems) was prepared as above. Primers (10 µM) were determined to amplify equally efficiently across a broad range of template dilutions by standard curve (listed in Table S1). The comparative C_T_ method was used to quantify targets; C_T_ values were normalized to *β-actin* levels in each tissue and expressed relative to the indicated target in the indicated tissues. Experiments were conducted at least four times for each primer set, with cycling parameters as follow: 50°C, 2 min; 95°C, 10 min; [95°C, 15 s; 60°C, 1 min] X 40 cycles. For initial experiments, where primer efficiencies were being determined, dissociation curves and –RT controls were included, indicating the specificity of amplification and lack of DNA contamination in template preparations, respectively (data not shown). Alternative splicing variants posed a problem in primer design for the *NAIP^ERV-P^* and *NAIP^Sg^* targets. For *NAIP^ERV-P^* we quantified only one of the variants and estimated that it accounted for ∼40% of all total LTR-derived transcripts, as before [15]. For *NAIP^Sg^*, we designed primers spanning exon junctions of both isoforms and combined their proportions.

### Generation of constructs

Placental genomic DNA was obtained from the laboratory of Dr. P. Medstrand (Lund University) and subsequently used to PCR amplify the *NAIP* promoter regions and open reading frame (ORF). *Promoter constructs.* Testis-specific LTR (or NAIP^ERV-P^), the ubiquitous NAIP^full^, and the *Alu*-derived NAIP^Sg^ and NAIP^Jb^ promoters were amplified by PCR using Phusion High Fidelity DNA Polymerase (Finnzymes) in an iCycler (BioRad) over 35 cycles, the primers used are listed in Table S1. The respective products are approximately 500 bp and centered on the transcription start sites. All primers possessed BglII and HindII recognition sites to facilitate directional cloning into a modified pGL3B vector described elsewhere [15]. Sequencing (McGill University and Génome Québec Innovation Centre) verified fidelity of amplified fragments.

#### Expression vector

The preserved ORF deriving from *NAIP^Sg^* and *NAIP^Jb^* transcripts was amplified by Phusion High Fidelity DNA Polymerase (Finnzymes) from human testis cDNA (as described above) over 35 cycles, primer sequences are indicated in Table S1. The desired amplicon was isolated using the PureLink Quick Gel Extraction Kit (Invitrogen) and subsequently dATP-tailed with Taq DNA Polymerase (Invitrogen) to facilitate cloning into the pGEM-T vector (Promega). Sequencing not only confirmed that the ORF was cloned error-free, but also that *NAIP2* is expressed, in addition to *NAIP^full^*, on account of a single representative nucleotide difference. *Xho1* and *Nco1* recognition sites incorporated into primers were utilized to subclone the sequenced ORF into the CTV 211 hemagglutinin (HA) epitope-bearing mammalian expression vector, generously provided by Dr. R. Kay (Terry Fox Laboratory). All vectors were amplified in *E. coli* DH5α and purified using the Nucleobond AX (Clontech) maxi prep kit, and quantified using the Qubit fluorometer (Invitrogen).

### Cell culture and transient transfection

HeLa, NTera2D1, LNCaP, and Jeg3 cells were cultured in DMEM (Stem Cell Technologies) and PC3 cells in RPMI 1640 (Stem Cell Technologies), and incubated at 37° and 5% CO_2_. All media formulations were supplemented with 10% Fetal Bovine Serum (Invitrogen) and maintained in penicillin/streptomycin, except when undergoing transfection experiments. Prior to transfection of promoter constructs cells were seeded at 10^5^ cells/well, or 2×10^5^ cells/well for NTera2D1, in a 24-well dish overnight. Lipofectamine 2000 (Invitrogen) was used to transfect the indicated cells with the indicated vectors according to manufacturer's specifications. Approximately 6-8 hours post-transfection cells were washed with PBS (Stem Cell Technologies) and fresh complete media was added to allow for production of the reporter for an additional ∼24 hours. The HA∶NAIP expression vector, was transiently transfected into HeLa, PC3, and NTera2D1 cells using Metafectene (Biontex) as recommended by the manufacturer.

### Reporter gene assays

Prior to lysis, cells were washed with PBS, processed, then analyzed for firefly and *Renilla* luciferase activity using the Dual Luciferase Reporter Assay System (Promega) as indicated by the manufacturer. All values were standardized to the *Renilla* luciferase internal control to normalize for transfection efficiency, then expressed relative to the modified promoterless pGL3-Basic vector.

### Western blotting

Cells were grown in 10 cm dishes as indicated above. The human PC3, NTera2D1, and HeLa cell lines were selected to screen for NAIP proteins based on preliminary RT-PCR findings (data not shown). Cells transfected with the expression vector encoding the *Alu*-derived *NAIP* ORF or untransfected controls were harvested by either scraping or trypsinization following two washes with cold PBS. Cell pellets were obtained by centrifugation and resuspended in RIPA (150 mM NaCl; 1% NP-40; 0.5% sodium deoxycholate; 0.1% SDS; 50 mM Tris, pH8) and NP40 (150 mM NaCl; 1% NP-40; 50 mM Tris, pH8) lysis buffers supplemented with a protease inhibitor cocktail (Roche), and subsequently quantified using the Qubit Fluorometer (Invitrogen). Hemagglutinin epitope signal was easier to detect in NP40 lysates, while RIPA provided clearer results for the NAIP-specific antibody. Bi-phased gels containing TEMED and APS (4% stacking, 9% separating) were used to resolve total cellular protein in electrophoresis running buffer (10×: 25 mM Tris; 192 mM glycine; 0.1% SDS). Subsequently, separated proteins were transferred using a Hoefer TE 22 tank transfer unit (Amersham Biosciences) onto Immobilon-P PVDF membrane (Millipore) in fresh transfer buffer (25 mM Tris, 192 mM glycine, 10% methanol, 0.1% SDS). To assess NAIP protein isoforms in primary human tissues an IMB-103-50 Instablot membrane was purchased from Imgenex (San Diego). Blocking of all membranes was performed in 5% reconstituted skim milk powder under constant agitation at 4° overnight. The following morning, blocking solution was replaced and fresh primary antibodies were applied at 1∶1000 NAIP (Abcam), 1∶3500 Actin (Sigma), and 1∶3500 HA (BAbCO) for one hour at room temperature under constant agitation. Washes were carried out with TBS-T (10×: 20 mM Tris;1.4 M NaCl;1% Tween-20) at room temperature in 5 minute intervals, no more than five times. Secondary antibody was diluted in fresh TBST and 1% blocking solution to a final concentration of 1∶100 000, and incubated for one hour at room temperature under constant agitation. Washes were conducted as above. Proteins were detected using the Enhanced Chemiluminescence Kit (Perkin Elmer) and Kodak BioMax Film and cassettes (Kodak). Where necessary the Instablot was stripped with 0.2 M NaOH, all other membranes were cleared by an acidic strip solution (25 mM glycine-HCl pH2, 1% SDS).

### Computational tools

#### Dot plots

Analysis of the underlying DNA sequence of 5q13.3 was performed to better understand the exons mapping to particular *NAIP* copies. DNA sequences were obtained from the UCSC Human Genome Browser March 2006 (hg18) assembly [34]. The genomic sequence of *NAIP^full^* (chr5:70,298,269-70,360,000) was used to assess exon architecture of the remaining copies: *NAIP1* (chr5:70,425,120-70,469,539); *NAIP2* (chr5:69,424,009-69,495,811); and *ψNAIP1* and *2* (chr5:69,780,634-69,828,298; 68,921,612-68,967,595). Indicated sequences were compared using the web-based jdotter (http://athena.bioc.uvic.ca/workebnch.php?tooljdotter&db=). *Sequence Analysis*. Sequenced clones were uploaded, managed, and analyzed in the SDSC Biology Workbench (http://workbench.sdsc.edu). Precise mapping of the clones to the human genome was completed using the BLAT tool in the UCSC Genome Browser [34]. *ORF prediction.* Sequences of interest were scanned for open reading frames using NCBI's ORF Finder, and subsequent analysis of encoded domains was completed with BLASTP.

## Supporting Information

Figure S1 Homology of human *NAIP* copies. Dot plots were performed to better understand the exon architecture of each *NAIP* copy. The *NAIPfull* copy in the 2006 assembly of the human genome (70,298,269–70,360,000) was compared to the genomic sequence underlying the other *NAIP* copies (as indicated). The coordinates of tested sequences are shown.
(4.44 MB TIF)

Figure S2 Unequal levels of *NAIP* 5′ and 3′ transcription. Semi-quantitative RT-PCR was performed at a low cycle number across a panel of human tissues to determine the levels of *NAIP* 5′ and 3′ transcription. Red arrowheads indicate localization of the primers used in this experiment, and are shown relative to a diagram of *NAIPfull*, at bottom.
(7.43 MB TIF)

Figure S3 Analysis of *NAIPfull* transcription. A) *NAIPfull*-associated TSS are shown (bent arrows) as previously described: i and ii [15]; and iii [36]. Black boxes indicate exons, and labeled boxes represent LTRs (shaded) and SINEs (speckled). Colored arrowheads indicate tiled primers used to better understand the TSS associated with *NAIPfull* transcription in THP1 cells [36]. B) Tiled-primer experiments in the indicated primary human tissues and cell lines. The primers used are color-coded with those shown above (A). Primary tissues were Southern blotted to increase resolution, using a radio-labeled oligonucleotide specific for a region of exon 1 common to all isoforms.
(10.09 MB TIF)

Figure S4 Sequence analysis underlying *NAIP* transcription start sites for the novel *NAIPSg* (A), *NAIPJb* (B), and *NAIPGUSBP1* (C) regulatory regions. cDNA sequence is shown in capitalized letters and the underlying genomic DNA (gDNA) is shown in lower case. Subscript numbers associated with green (Alu) or purple (L1) font in the gDNA track denote positions along the relevant transposable element. All discovered transcription start sites are indicated in black bold-face, and superscript numbers in B and C represent the number of clones arising from the particular position. Vertical dashed lines in A, B, and C represent exon junctions, and slight extension of gDNA underlying exon junctions indicates the appropriate splice donor and acceptor sites. Splicing of *NAIPJb* clones does not occur and transcription proceeds through intervening intron 9 into exon10. Red bold-faced letters in A and B indicate sites of RNA-editing. Potential regulatory motifs are shown relative to the lower case genomic DNA sequences as follow: TATA box - italics; Initiator sequences - overlines; Downstream promoter elements - underlines [39]; yellow, light blue, and dark blue shading denote estrogen response element, retinoic acid response element, and AP-1 binding motifs, respectively [13].
(0.05 MB DOC)

Figure S5 Broad transcription of novel *NAIP* isoforms. RT-PCR was performed to determine the breadth of expression of NAIP from the *Alu* and *GUSBP1* 3′ UTR-contained TSS, represented by bent arrows. Color-coded arrows indicate the primers used: expression from *NAIPSg* is indicated by blue arrows and box; expression from *NAIPGUSBP1* is indicated by purple arrows and box; and expression from *NAIPJb* is indicated by orange arrows and box. No splicing is observed between the *Alu*Jb transcription start site and the adjacent downstream exon; +/− RT controls indicate low, or no, contamination of genomic DNA. Diagrams are not drawn to scale.
(7.82 MB TIF)

Figure S6 NAIP protein sequence and encoded domains. The protein sequence of NAIP^full^ is shown, and exon boundaries are indicated by numbers above circled arrows. Potential downstream in-frame initiation codons are indicated in red font, and the surrounding nucleotide sequence is shown beneath, with ‘atg’ in boldface. Underlines represent start codons with a sequence context in general agreement with derived consensi [42], [43]. The stop codon is denoted by an asterisk. Yellow, purple, and green highlighting indicates BIR, NBD, and LRR domains, respectively.
(0.03 MB DOC)

Table S1 Primers used in this report. A list of all primers used throughout this investigation is sectioned according to the general application for which they were designed. Associated with each primer is the sequence, the T_m_ at which it was utilized, as well as a note specifying its particular application.
(0.07 MB XLS)
