# Supplementary figures and images for: A Novel Protein Isoform of the Multicopy Human NAIP Gene Derives from Intragenic Alu SINE Promoters
Source: PLoS One. 2009 Jun 2;4(6):e5761. doi: 10.1371/journal.pone.0005761 (PMC2685007; doi:10.1371/journal.pone.0005761)

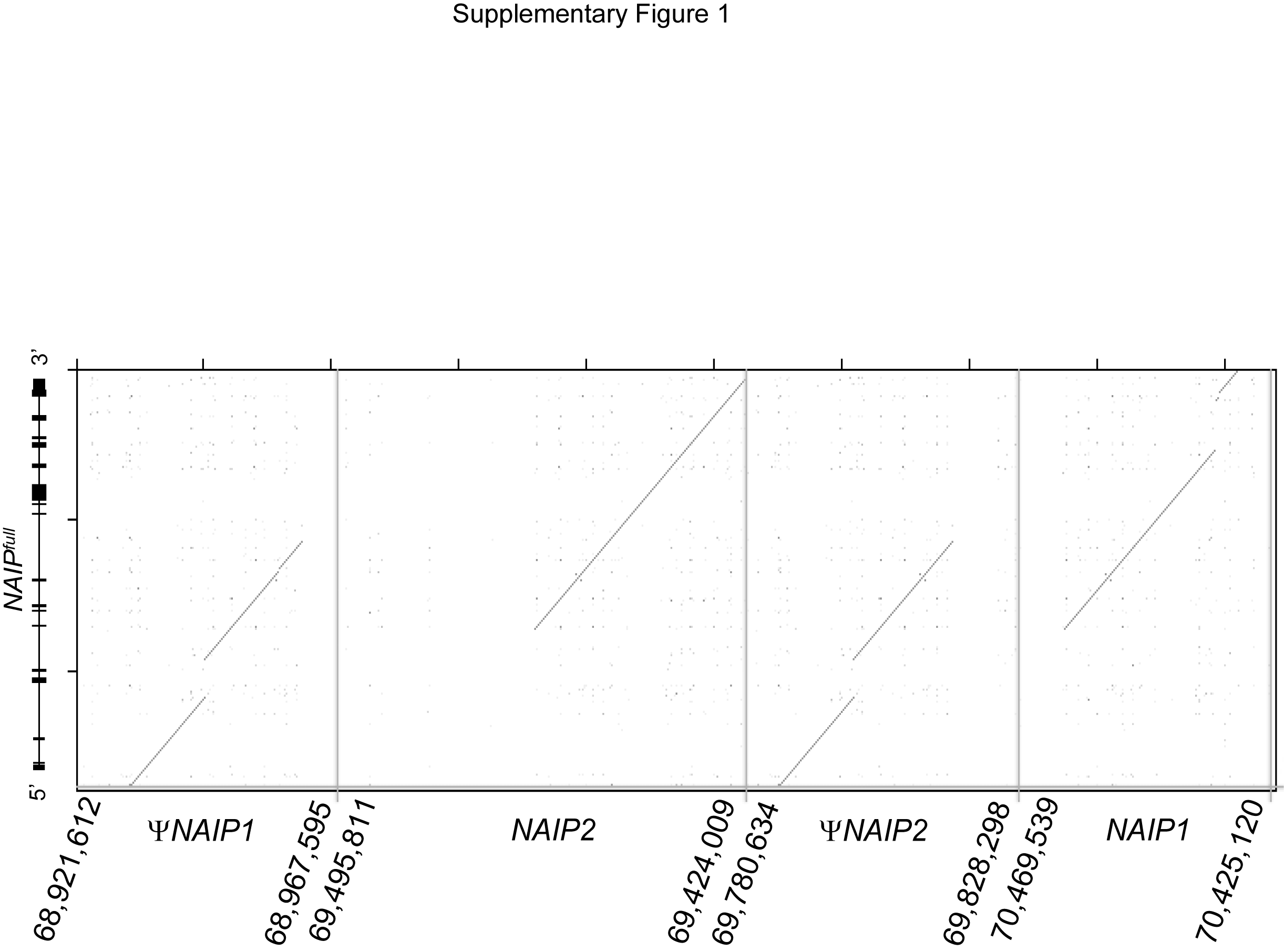

Supplement: Figure S1 — Homology of human NAIP copies. Dot plots were performed to better understand the exon architecture of each NAIP copy. The NAIPfull copy in the 2006 assembly of the human genome (70,298,269–70,360,000) was compared to the genomic sequence underlying the other NAIP copies (as indicated). The coordinates of tested sequences are shown. (4.44 MB TIF) [file pone.0005761.s001.tif]

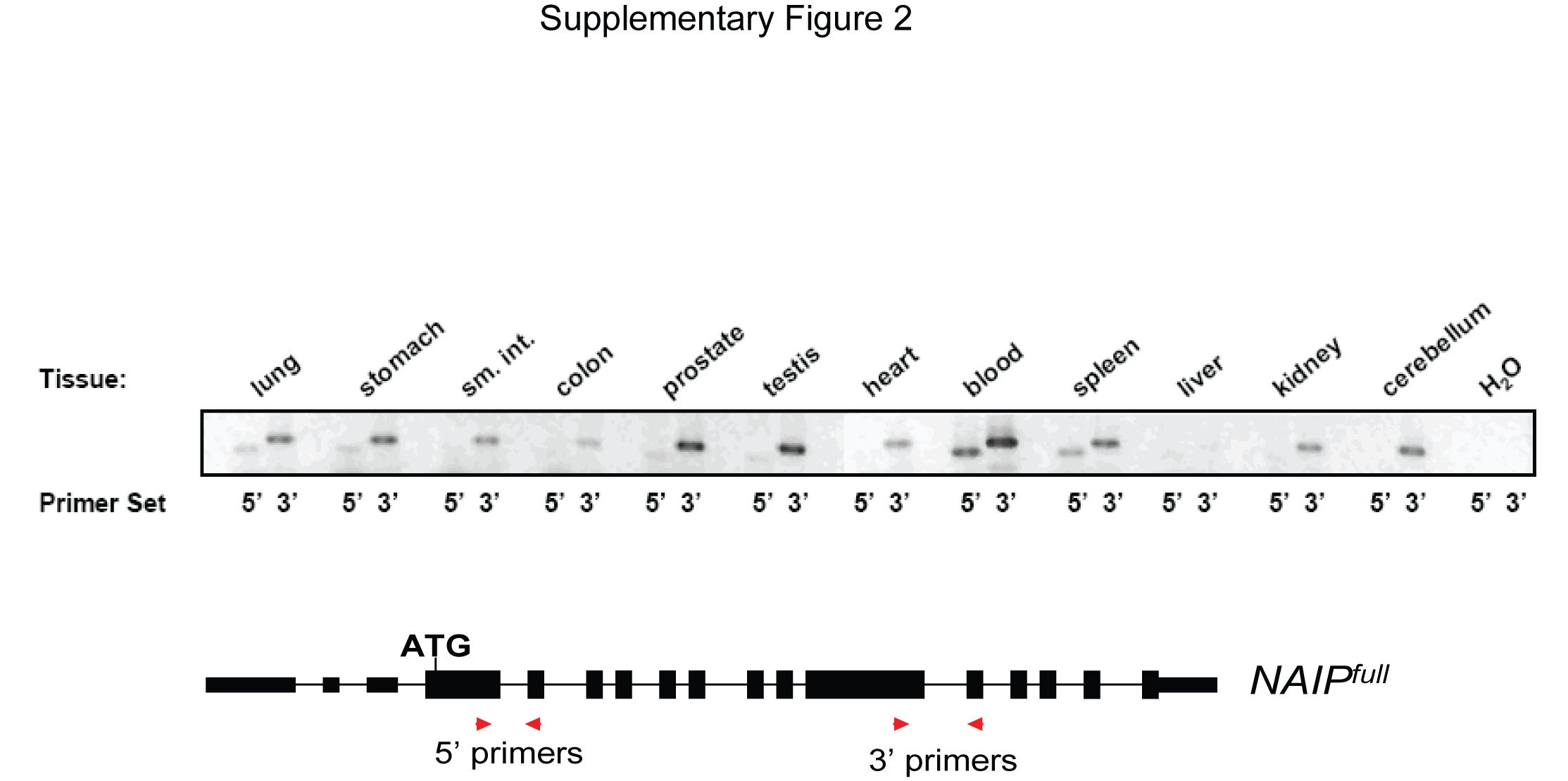

Supplement: Figure S2 — Unequal levels of NAIP 5′ and 3′ transcription. Semi-quantitative RT-PCR was performed at a low cycle number across a panel of human tissues to determine the levels of NAIP 5′ and 3′ transcription. Red arrowheads indicate localization of the primers used in this experiment, and are shown relative to a diagram of NAIPfull, at bottom. (7.43 MB TIF) [file pone.0005761.s002.tif]

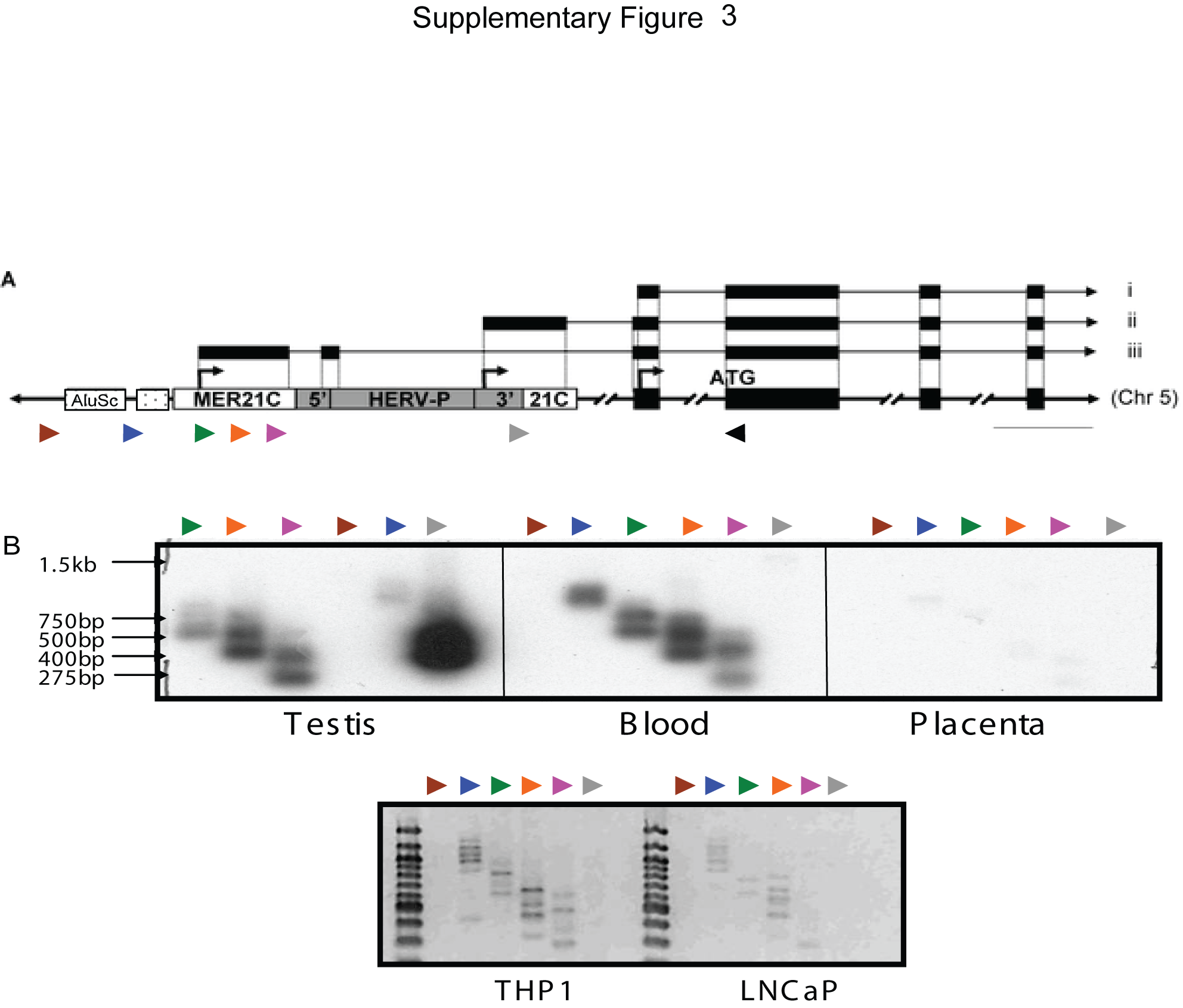

Supplement: Figure S3 — Analysis of NAIPfull transcription. A) NAIPfull-associated TSS are shown (bent arrows) as previously described: i and ii [15]; and iii [36]. Black boxes indicate exons, and labeled boxes represent LTRs (shaded) and SINEs (speckled). Colored arrowheads indicate tiled primers used to better understand the TSS associated with NAIPfull transcription in THP1 cells [36]. B) Tiled-primer experiments in the indicated primary human tissues and cell lines. The primers used are color-coded with those shown above (A). Primary tissues were Southern blotted to increase resolution, using a radio-labeled oligonucleotide specific for a region of exon 1 common to all isoforms. (10.09 MB TIF) [file pone.0005761.s003.tif]

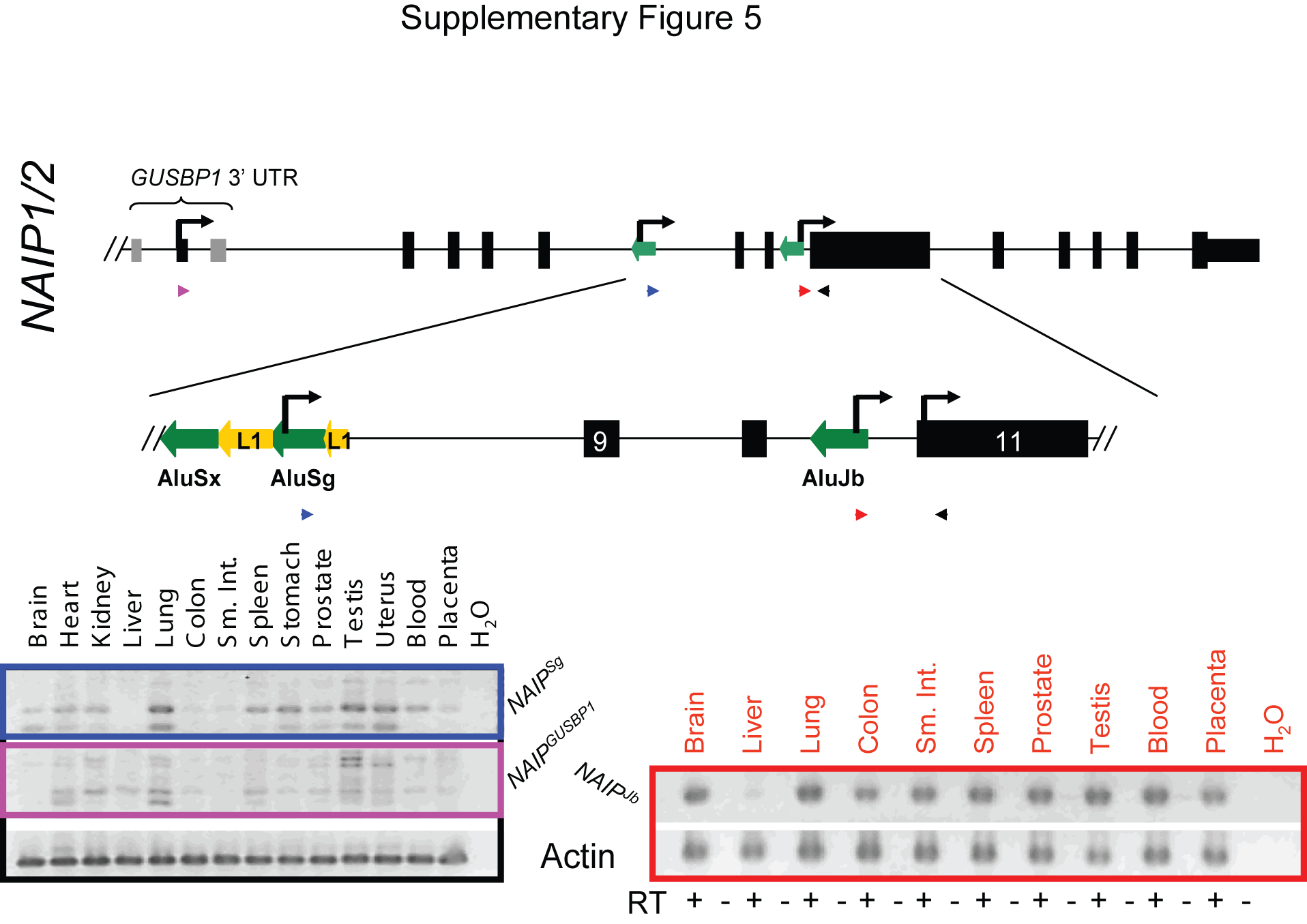

Supplement: Figure S5 — Broad transcription of novel NAIP isoforms. RT-PCR was performed to determine the breadth of expression of NAIP from the Alu and GUSBP1 3′ UTR-contained TSS, represented by bent arrows. Color-coded arrows indicate the primers used: expression from NAIPSg is indicated by blue arrows and box; expression from NAIPGUSBP1 is indicated by purple arrows and box; and expression from NAIPJb is indicated by orange arrows and box. No splicing is observed between the AluJb transcription start site and the adjacent downstream exon; +/− RT controls indicate low, or no, contamination of genomic DNA. Diagrams are not drawn to scale. (7.82 MB TIF) [file pone.0005761.s005.tif]
