# Supplementary material for: A Novel Protein Isoform of the Multicopy Human NAIP Gene Derives from Intragenic Alu SINE Promoters
Source: PLoS One. 2009 Jun 2;4(6):e5761. doi: 10.1371/journal.pone.0005761 (PMC2685007; doi:10.1371/journal.pone.0005761)
Supplement: Figure S4 — Sequence analysis underlying NAIP transcription start sites for the novel NAIPSg (A), NAIPJb (B), and NAIPGUSBP1 (C) regulatory regions. cDNA sequence is shown in capitalized letters and the underlying genomic DNA (gDNA) is shown in lower case. Subscript numbers associated with green (Alu) or purple (L1) font in the gDNA track denote positions along the relevant transposable element. All discovered transcription start sites are indicated in black bold-face, and superscript numbers in B and C represent the number of clones arising from the particular position. Vertical dashed lines in A, B, and C represent exon junctions, and slight extension of gDNA underlying exon junctions indicates the appropriate splice donor and acceptor sites. Splicing of NAIPJb clones does not occur and transcription proceeds through intervening intron 9 into exon10. Red bold-faced letters in A and B indicate sites of RNA-editing. Potential regulatory motifs are shown relative to the lower case genomic DNA sequences as follow: TATA box - italics; Initiator sequences - overlines; Downstream promoter elements - underlines [39]; yellow, light blue, and dark blue shading denote estrogen response element, retinoic acid response element, and AP-1 binding motifs, respectively [13]. (0.05 MB DOC) [file pone.0005761.s004.doc]

Supplementary Figure 4a

gDNA – ttccatctgctatcattttcct*tttaaa*tgatgtgcttcctttcac_2101305_ttttttttttttttt

clone1- **T**TTTTTCTTTGAGATGGAGTCTTGCTCTGTCTCCTAGGCTGGAGTTCAGCGCCACGATCTC

clone2 - **T**TTTTTTTTTGAGATGGAGTCTTGCTCTGTCTCCTAGGCTGGAGTTCAGCGCCACGATCTC

clone3 - **T**TTTTTTTTTGAGATGGAGTCTTGCTCTGTCTCCTAGGCTGGAGTTCAGCGCCACGATCTC

clone4 - **---------**---**G**ATGGAGTCTTGCTCTGTCTCCTAGGCTGGAGTTCAGCGCCACGATCTC

gDNA -ttttttttttgagatggagtcttgctctgtctcctaggctggagttcagcgccacgatctc

clone1 -GGCTGACTGCA**G**CCTCTGCCTCCCGGGTTCAAGTGATTCTCCTGCCTCAACCTCCTGAGTA

clone2 - GGCTGACTGCA**G**CCTCTGCCTCCCGGGTTCAAGTGATTCTCCTGCCTCAACCTCCTGAGTA

clone3 - GGCTGACTGCAACCTCTGCCTCCCGGGTTCAAGTGATTCTCCTGCCTCAACCTCCTGAGTA

clone4 - GGCTGACTGC**G**ACCTCTGCCTCCCGGGTTCA**GA**TGATTCTCCTGCCTCA**G**CCTCCTGAGTG

gDNA -ggctgactgcaacctctgcctcccgggttcaagtgattctcctgcctcaacctcctgagta

clone1 – GCT**G**GGATT**G**CAG TTGTCCATTTCTCCAAAATATGAAGTCCTCTGCGGAAGTGACTC…

clone2 – GCT**G**GGATT**G**CAG TTGTCCATTTCTCCAA**G**ATATGAAGTCCTCTGCGGAAGTGACTC…

clone3 – GCTAGGATT**G**CAG TTGTCCATTTCTCCAAAATATGAAGTCCTCTGCGGAAGTGACTC…

clone4 – GCT**G**GGATT**G**CAG TTGTCCATTTCTCCAAAATATGAAGTCCTCTGCGGAAGTGACTC…

gDNA – gctaggattacaggtagttgtccatttctccaaaatatgaagtcctctgaggaagtgactc…

gcccaccaccatgccaggctaatttttgtatttttagtagagatagagtttcaccatgttggccaggctggtcttgaactcctgacctcatgatctgcctgcctcagcctcccaaagtgctgggattacaggggtgaaccactgtgcctggtc_1_…

Supplementary Figure 4b

gDNA - ^5’^…_163_accacacctggctaatttttg*tatt*ttttg*taga*gacagggttttgccatgttggccag

clones - **G^1^**CTGGTCTTGAACTCCTAGGCTCAAGCAATTCGCCTGCCTCGGTCTCCCACAGTGCTGGG

gDNA - g^-^ctggtcttgaactcctaggctcaagcaattcgcctgcctcggtctcccacagtgctggg

clones - ATTACAGGCATGAGTCAC**T^5^**TTGCCTGGCC^-^TCTTTCCTGA**G^1^**ATGCATGG**T^1^**GCTT**G**TGAT

gDNA - attacaggcatgagtcact^-^ttgcctggcc_1_tctttcctgag^-^atgcatggt^-^gcttatgat

clones - AAGCACACATTATGTCTAGGTCCCTGCTTCAAGTGTGGCACTTTGGACACATGCTTCCCAC

gDNA - aagcacacattatgtctaggtccctgcttcaagtgtggcactttggacacatgcttcccac

clones - ATTCCGATTTTGTGCCAAAACCTATGAGATGATCGCAATGTGGGAATCATGGATGGCTGTG

gDNA - attccgattttgtgccaaaaggtatgagatgatcgcaatgtgggaatcatggatggctgtg

clones - GAAAATCCTAACACATTCATAGTAGACAGGCAGAATCATGGA**A^1^**TGAAAAGGCATGGCGTT

gDNA - gaaaatcctaacacattcatagtagacaggcagaatcatggaa^-^tgaaaaggcatggcgtt

clones - CAGACTGAGGGAGATGTGACTATGAATCCCTGTTGTGCCCCC**C^1^**TTTC**T^1^**TTCTCTCCACAG

gDNA - cagactgagggagatgtgac*tatgaa*tccctgttgtgcccccc^-^tttct^-^ttctctccacag

clones - AAATGGCACAGGGTGAAGCCCAGTGGTTTCAAGAGGCAAAGAATCTGAATGAGCAGCTGA

gDNA - aaatggcacagggtgaagcccagtggtttcaagaggcaaagaatctgaatgagcagc_Exon10…_^3’^

Supplementary Figure 4c

gDNA …ac*taataa*aaacataggaatcaaacagacg*aataa*aatgctccctgcccacacagccat

NAIP1/2 **A^2^**A**C^1^**ATCCTATGGCCCTGAAAATAGTTCTGCCAAGCTGCGTGCAGTGGACACTTCACGGCT

gDNA a^-^ac^-^atcctatggccctgaaaatagttctgccaagctgcgtgcagtggacacttcacggct

NAIP1/2 GGCACAGGAGAGATCACAAGGCCTTGCTCAATTTCATCAAAAGTGTTAAAGCGACTCAGC

gDNA ggcacaggagagatcacaaggccttgctcaatttcatcaaaagtgttaaagcgactcagc

NAIP1/2 AGATTGTGAAGCACAAGTGGAAGCTGATAATTGGTGTTTCTTACAAATCAACGGCTTGTC

gDNA agattgtgaagcacaagtggaagctgataattggtgtttcttacaaatcaacggcttgtc

NAIP1/2 TCCACACATCAAG ATGTGAATTTCTTCGGAGTAAGAAATCCTCAGAGGAAATTA

gDNA tccacacatcaaggt……agatgtgaatttcttcggagtaagaaatcctcagaggaa…_Exon4_
