# Supplementary material for: A Novel Protein Isoform of the Multicopy Human NAIP Gene Derives from Intragenic Alu SINE Promoters
Source: PLoS One. 2009 Jun 2;4(6):e5761. doi: 10.1371/journal.pone.0005761 (PMC2685007; doi:10.1371/journal.pone.0005761)
Supplement: Figure S6 — NAIP protein sequence and encoded domains. The protein sequence of NAIPfull is shown, and exon boundaries are indicated by numbers above circled arrows. Potential downstream in-frame initiation codons are indicated in red font, and the surrounding nucleotide sequence is shown beneath, with ‘atg’ in boldface. Underlines represent start codons with a sequence context in general agreement with derived consensi [42], [43]. The stop codon is denoted by an asterisk. Yellow, purple, and green highlighting indicates BIR, NBD, and LRR domains, respectively. (0.03 MB DOC) [file pone.0005761.s006.doc]

Supplementary Figure 6

2 caa**atg**cgc

---MATQQKASDERISQFDHNLLPELSALLGLDAVQLAKELEEEEQKERAKMQKGYNSQMRSE

aaa**atg**gcc aaa**atg**cag

AKRLKTFVTYEPYSSWIPQEMAAAGFYFTGVKSGIQCFCCSLILFGAGLTRLPIEDHKRFHPDC

gag**atg**gcg

GFLLNKDVGNIAKYDIRVKNLKSRLRGGKMRYQEEEARLASFRNWPFYVQGISPCVLSEAGFVF

aaa**atg**agg

3 4

TGKQDTVQCFSCGGCLGNWEEGDDPWKEHAKWFPKCEFLRSKKSSEEITQYIQSYKGFVDIT

5 6 7

GEHFVNSWVQRELPMASAYCNDSIFAYEELRLDSFKDWPRESAVGVAALAKAGLFYTGIKD

cct**atg**gca

8 9

IVQCFSCGGCLEKWQEGDDPLDDHTRCFPNCPFLQNMKSSAEVTPDLQSRGELCELLETTSE

aat**atg**aag

10

SNLEDSIAVGPIVPEMAQGEAQWFQEAKNLNEQLRAAYTSASFRHMSLLDISSDLATDHLLGCD

aaa**atg**gca cac**atg**tct

LSIASKHISKPVQEPLVLPEVFGNLNSVMCVEGEAGSGKTVLLKKIAFLWASGCCPLLNRFQLV

gtc**atg**tgt

FYLSLSSTRPDEGLASIICDQLLEKEGSVTEMCVRNIIQQLKNQVLFLLDDYKEICSIPQVIGK

aaa**atg**tgc

ttt**atg**gtt

LIQKNHLSRTCLLIAVRTNRARDIRRYLETILEIKAFPFYNTVCILRKLFSHNMTRLRKFMVYF

ata**atg**act

GKNQSLQKIQKTPLFVAAICAHWFQYPFDPSFDDVAVFKSYMERLSLRNKATAEILKATVSSCG

tat**atg**gaa

acc**atg**tgc

ELALKGFFSCCFEFNDDDLAEAGVDEDEDLTMCLMSKFTAQRLRPFYRFLSPAFQEFLAGMRLI

ttg**atg**agc ggg**atg**agg

ELLDSDRQEHQDLGLYHLKQINSPMMTVSAYNNFLNYVSSLPSTKAGPKIVSHLLHLVDNKESL

ccc**atgatg**act

ENISENDDYLKHQPEISLQMQLLRGLWQICPQAYFSMVSEHLLVLALKTAYQSNTVAACSPFVL

cag**atg**cag tca**atg**gtt

QFLQGRTLTLGALNLQYFFDHPESLSLLRSIHFPIRGNKTSPRAHFSVLETCFDKSQVPTIDQD

YASAFEPMNEWERNLAEKEDNVKSYMDMQRRASPDLSTGYWKLSPKQYKIPCLEVDVNDIDVVG

cct**atg**aat tat**atg**gat**atg**cag

cta**atg**aca

QDMLEILMTVFSASQRIELHLNHSRGFIESIRPALELSKASVTKCSISKLELSAAEQELLLTLP

gat**atg**ctt

11

SLESLEVSGTIQSQDQIFPNLDKFLCLKELSVDLEGNINVFSVIPEEFPNFHHMEKLLIQISA

cat**atg**gag

12 13

EYDPSKLVKLIQNSPNLHVFHLKCNFFSDFGSLMTMLVSCKKLTEIKFSDSFFQAVPFVASL

ctc**atg**act**atg**ctt

14

PNFISLKILNLEGQQFPDEETSEKFAYILGSLSNLEELILPTGDGIYRVAKLIIQQCQQLHCL

15

RVLSFFKTLNDDSVVEIAKVAISGGFQKLENLKLSINHKITEEGYRNFFQALDNMPNLQELDI

aac**atg**cca

SRHFTECIKAQATTVKSLSQCVLRLPRLIRLNMLSWLLDADDIALLNVMKERHPQSKYLTILQK

aac**atg**tta gtc**atg**aaa

WILPFSPIIQK*
